# Supplementary material for: In vitro reconstitution of functional small ribosomal subunit assembly for comprehensive analysis of ribosomal elements in E. coli
Source: Commun Biol. 2020 Mar 25;3:142. doi: 10.1038/s42003-020-0874-8 (PMC7096426; doi:10.1038/s42003-020-0874-8)
Supplement: Supplementary file 1 — Supplementary Information [file 42003_2020_874_MOESM1_ESM.pdf]

Supplementary Figures

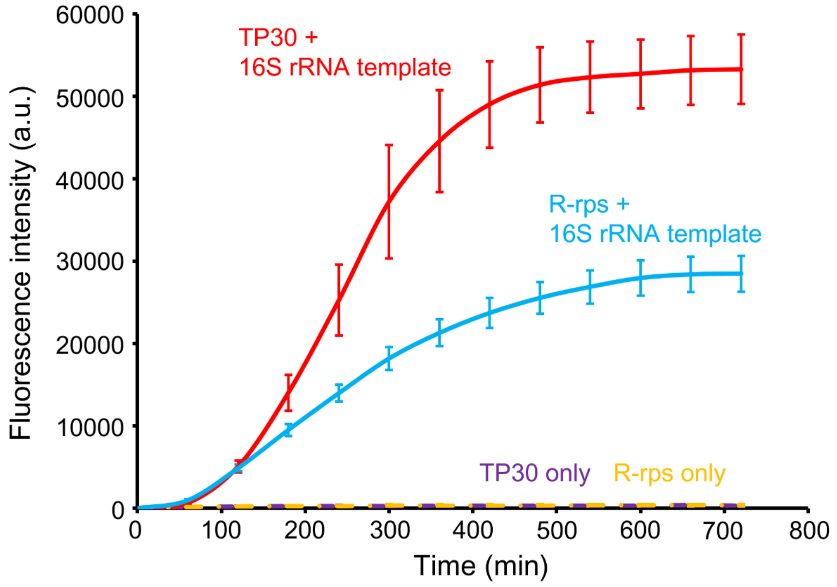

Supplementary Figure 1. Long time-course analysis of sfGFP synthesis in reactions with coupling of 16S rRNA transcription. R-rps respresents recombinant ribosomal proteins. Fluorescence intensities after subtracting the background intensity are shown. Error bars indicate standard deviation of at least triplicate measurements.

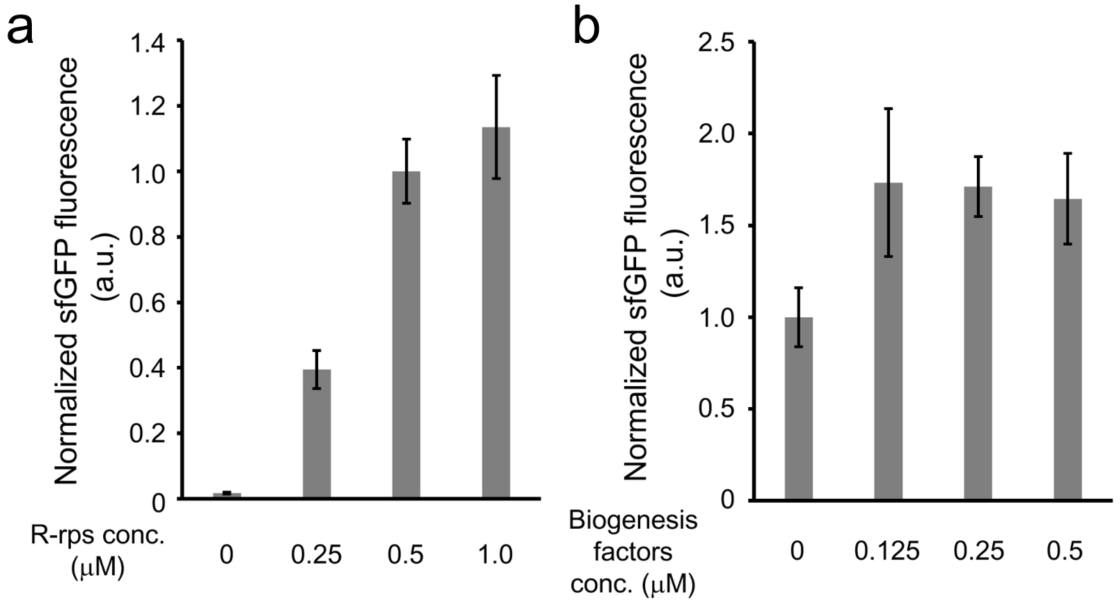

Supplementary Figure 2. Concentration dependencies of recombinant ribosomal protein mixtures (R-rps) and biogenesis factors on sfGFP synthesis in R-iSAT. Concentration dependencies of (**a**) R-rps and (**b**) biogenesis factors on sfGFP synthesis in R-iSAT were measured. Increase in sfGFP fluorescence after 4 h incubation was normalized by dividing by the average value of the control reaction. Error bars indicate standard deviation of at least triplicate measurements. Accordingly, we adopted 0.5 μM R-rps and 0.125 μM biogenesis factors throughout this study.

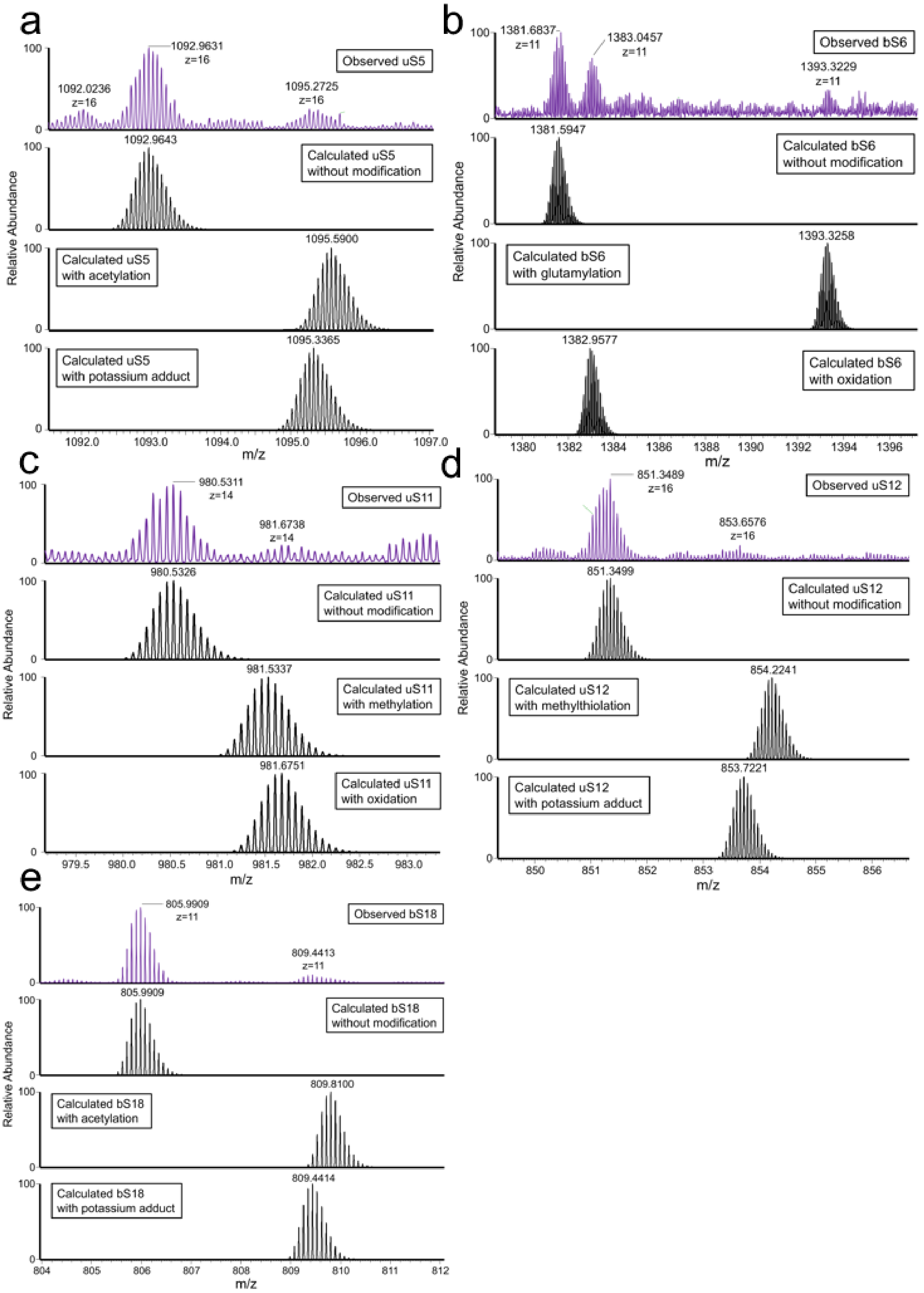

Supplementary Figure 3. Modification status of recombinant ribosomal proteins. Native MS analysis was performed for uS5 (**a**), bS6 (**b**), uS11 (**c**), uS12 (**d**), and bS18 (**e**), which are known to be post-translationally modified. Observed mass spectra of each

protein were shown in purple and calculated mass spectra of possible forms of each protein, according to the molecular weight and isotopic distribution, were shown in black.

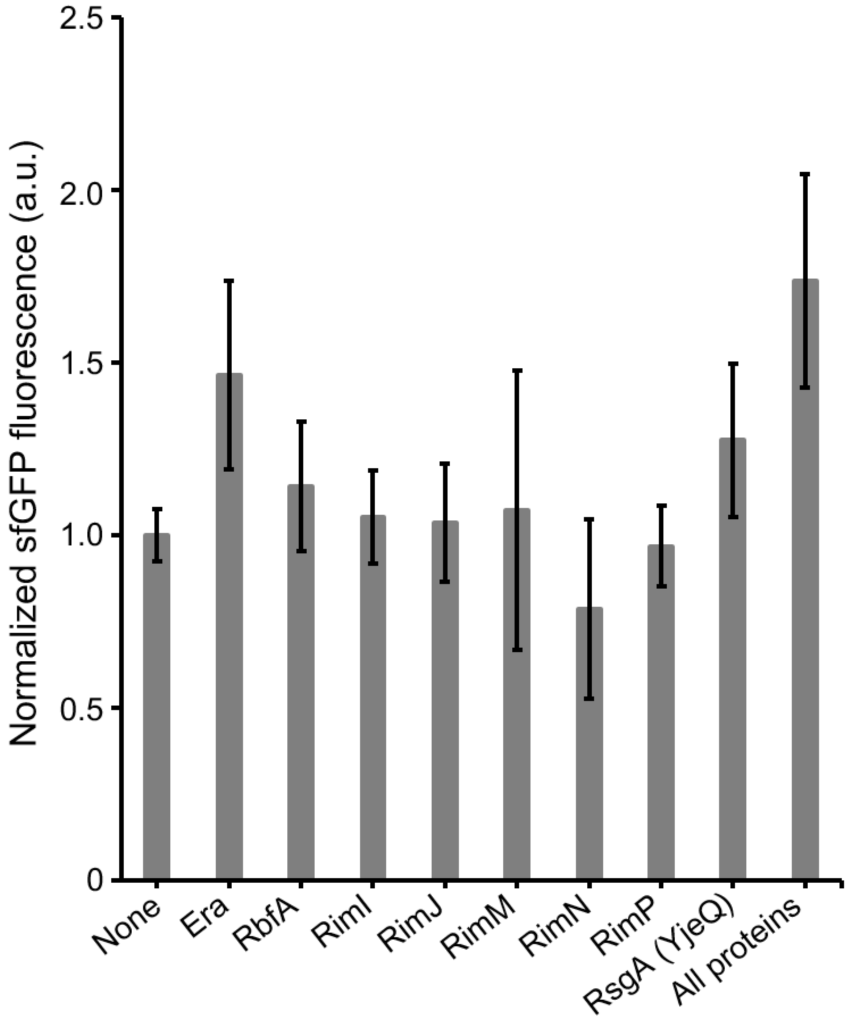

Supplementary Figure 4. Effects of ribosome biogenesis factors on R-iSAT. Increase in sfGFP fluorescence after 4 h incubation was normalized by dividing by the average value of the control reaction. Error bars indicate standard deviation of at least triplicate measurements.

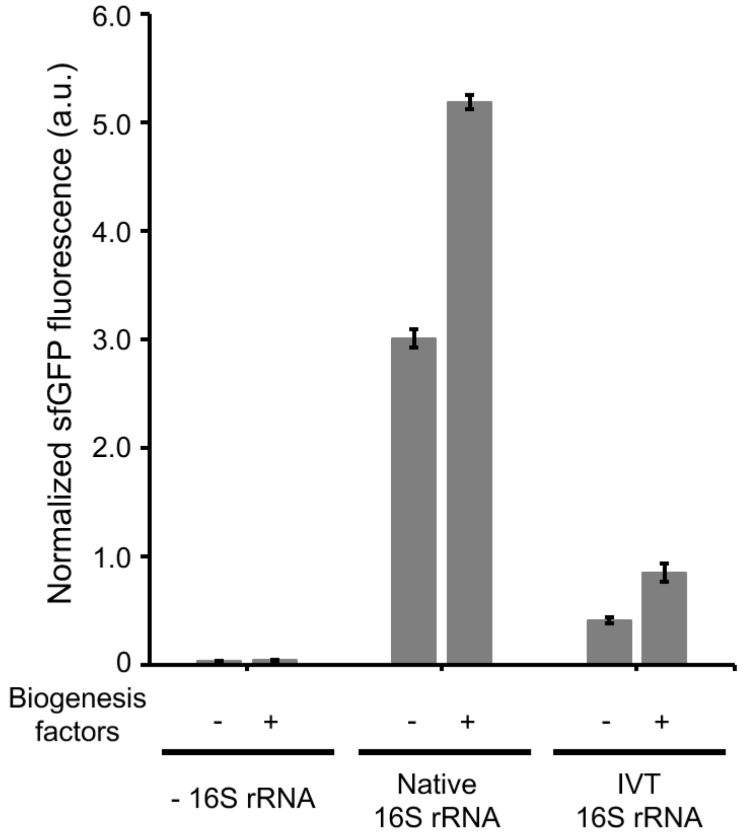

Supplementary Figure 5. Effects of ribosome biogenesis factors on reactions without coupled synthesis of 16S rRNA. Increase in sfGFP fluorescence after 4 h incubation was normalized by dividing by the average value of the control reaction. Error bars indicate standard deviation of at least triplicate measurements.

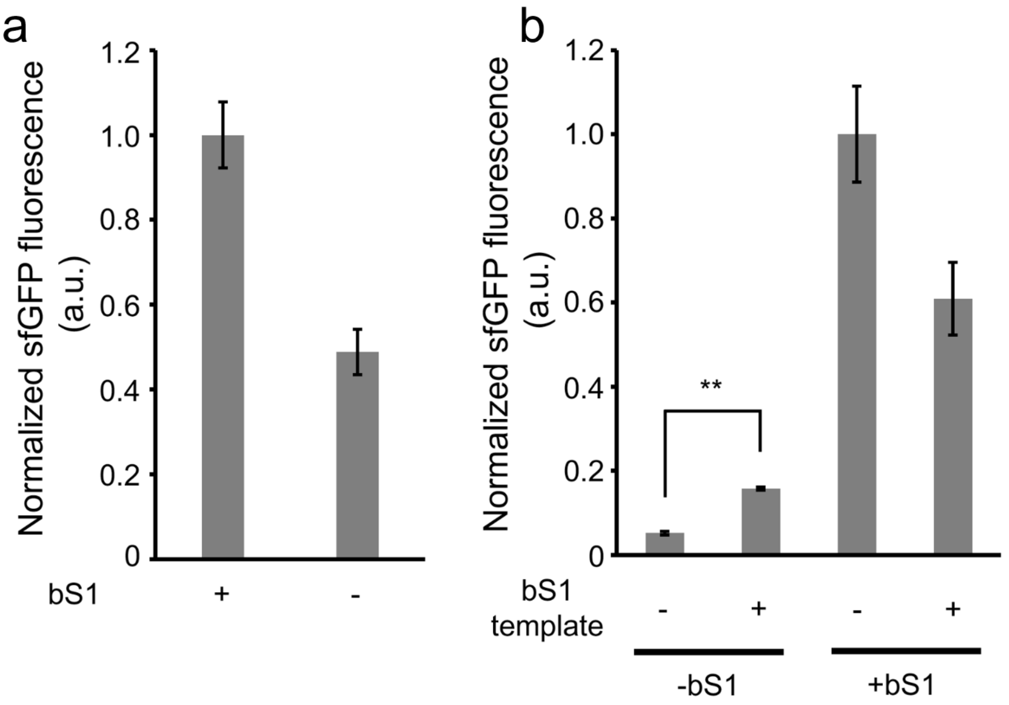

Supplementary Figure 6. Effects of bS1 removal and bS1 expression in R-iSAT. (**a**) Effect of bS1 removal from R-iSAT. (**b**) Effect of bS1 expression in R-iSAT in the absence and presence of bS1 protein. Increase in sfGFP fluorescence after 4 h incubation was normalized by dividing by the average value of the control reaction. Error bars indicate standard deviation of at least triplicate measurements. Double asterisk indicates that P values are less than 0.003. Welch's *t* test was applied between in the presence and absence of the rps template when bS1 was absent in the reaction mixtures.

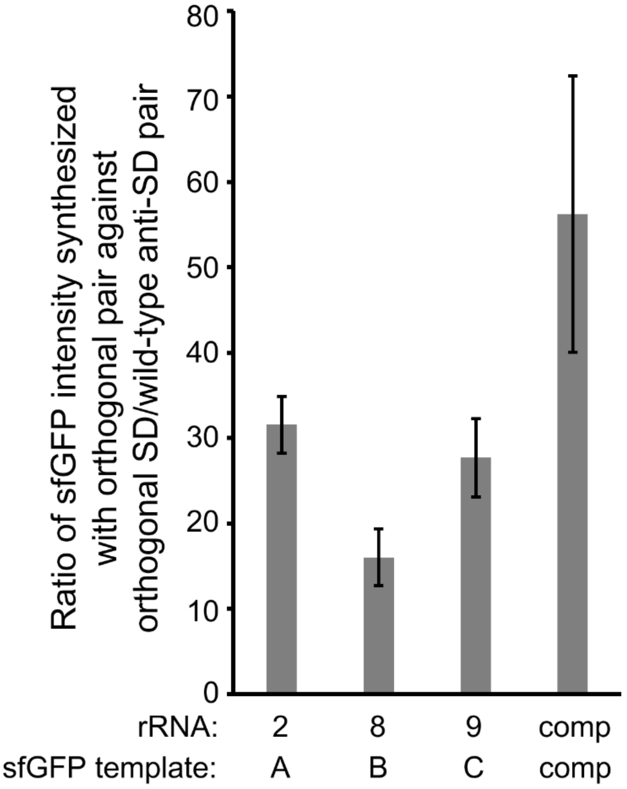

Supplementary Figure 7. Orthogonality against the wild-type ribosome. Ratio of sfGFP intensity synthesized with orthogonal pair against the intensity synthesized with wild-type ribosomes under the control of orthogonal SD sequences are shown. Error bars indicate standard deviation of at least triplicate measurements.

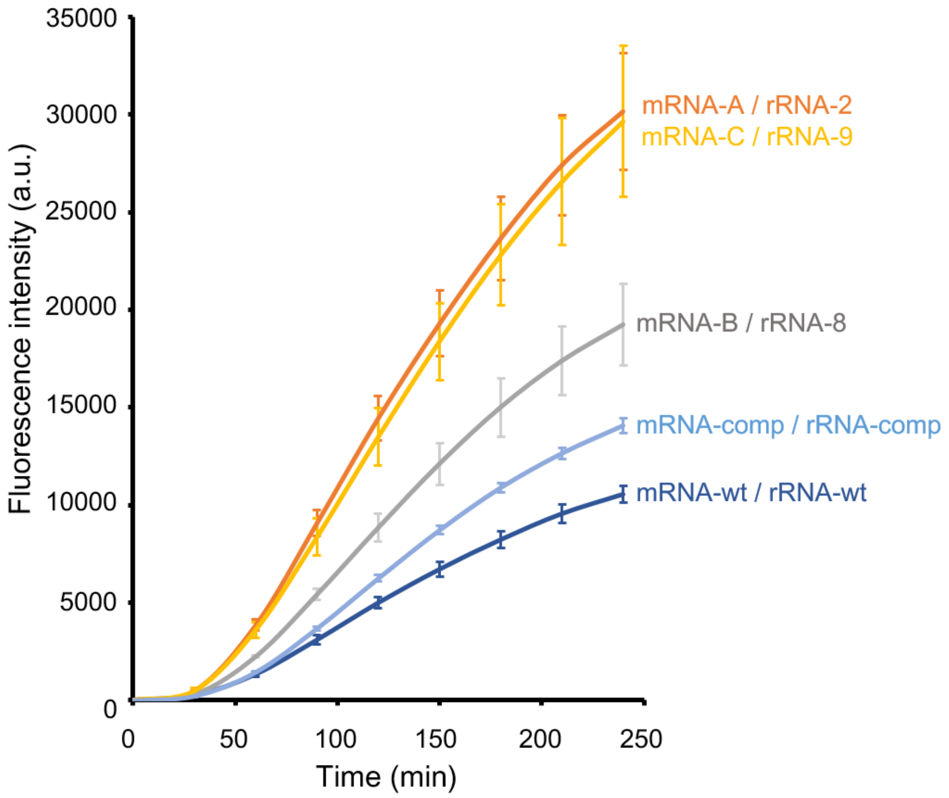

Supplementary Figure 8. Time-course analysis of sfGFP synthesis in R-iSAT with each SD/anti-SD pair. R-iSAT experiments were performed using SD/anti-SD pairs shown in **Fig. 3a**. Fluorescence intensities after subtracting the background intensity are shown. Error bars indicate standard deviation of at least triplicate measurements.

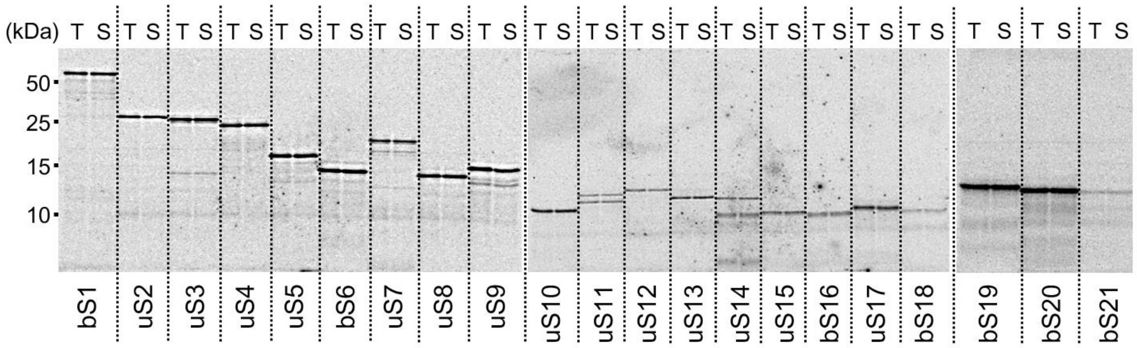

Supplementary Figure 9. Cell-free expression of ribosomal proteins in the PURE system. Radioisotope-labeled ribosomal proteins synthesized in the PURE system were analyzed by SDS-PAGE. Aggregate formation of the synthesized proteins were additionally assessed by centrifuging the total reaction mixtures (T) and then obtaining supernatant fractions (S).

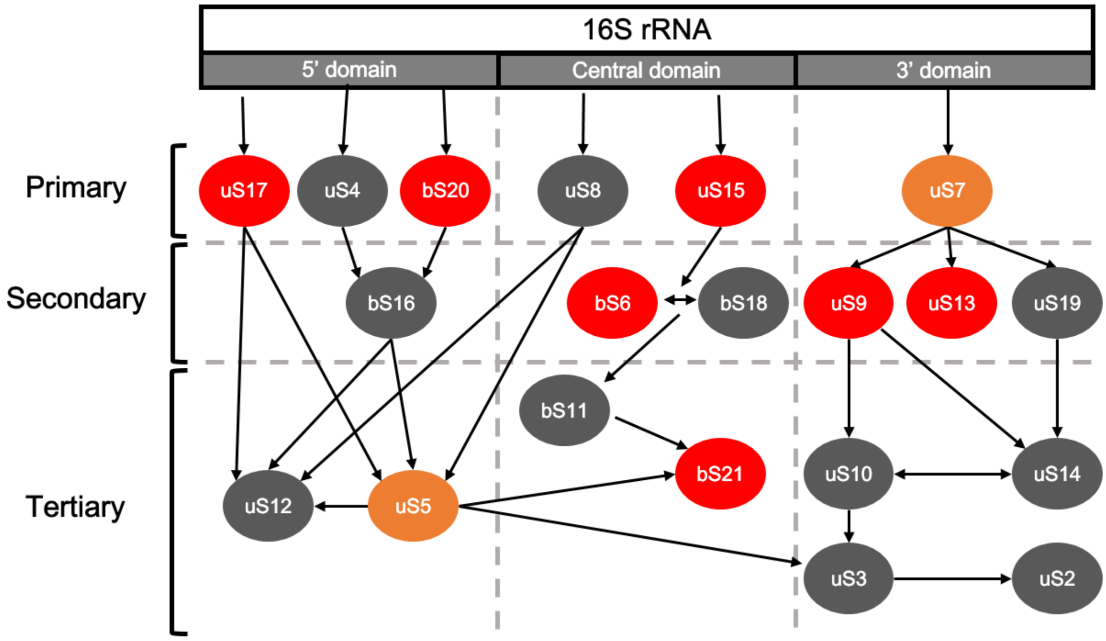

Supplementary Figure 10. Hierarchical assembly map of 30S subunits. Red circles represent non-essential ribosomal proteins, as revealed by genome deletion studies. Orange circles represent putative non-essential ribosomal proteins identified in this study.

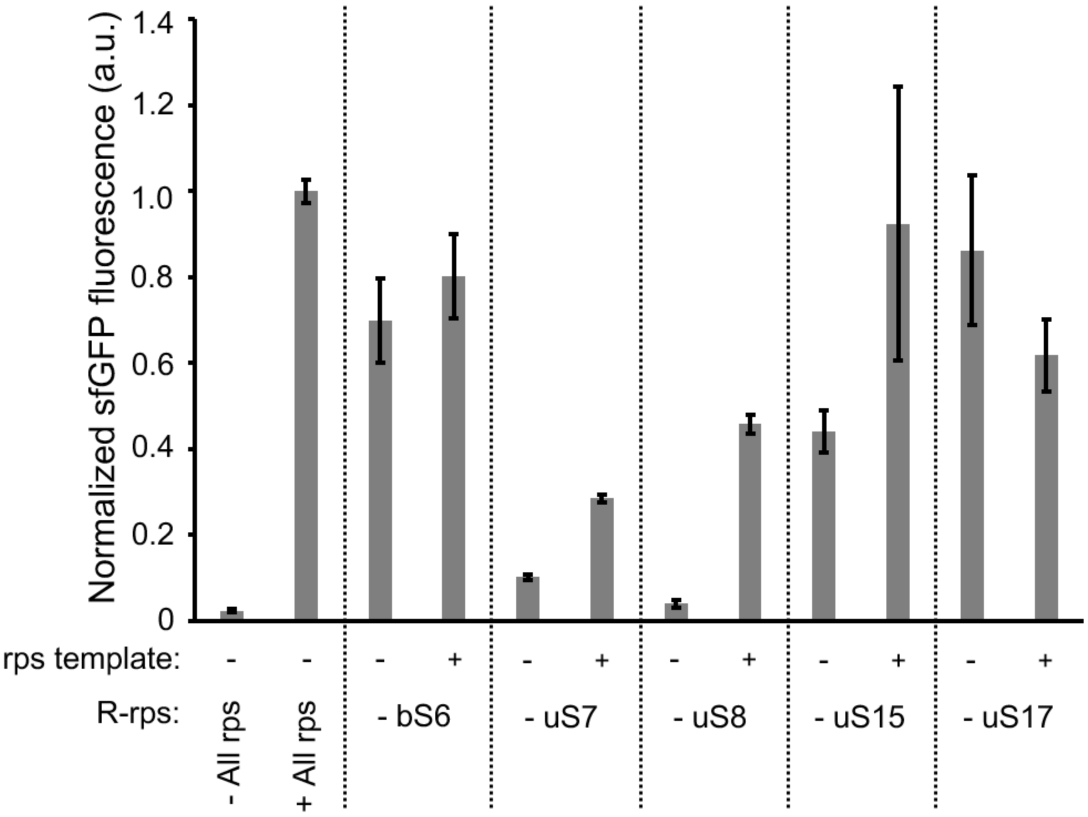

Supplementary Figure 11. Effects of pre-synthesis of ribosomal proteins. Reactions were separated into two steps: only the ribosomal protein synthesis was performed in the first step (2 h) in the presence of recombinant ribosomal proteins, and then template DNAs for 16S rRNA and sfGFP were added in the second step (4 h). The increase in sfGFP fluorescence after the 4-h incubation in the second step was normalized by dividing by the average value of the control reaction. Error bars indicate standard deviation of at least triplicate measurements.

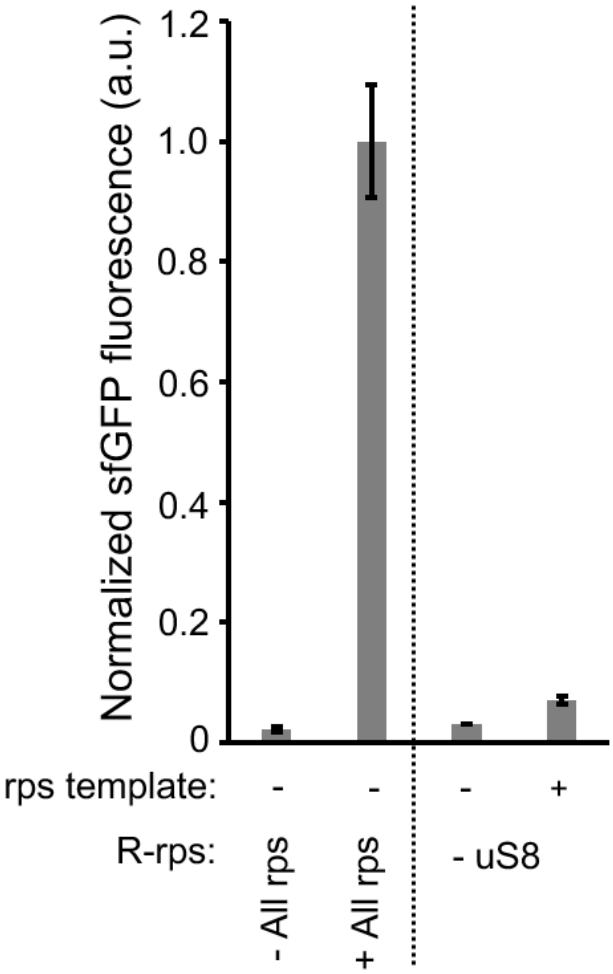

Supplementary Figure 12. Effects of pre-synthesis of both uS8 and 16S rRNA in the absence of recombinant ribosomal proteins. Reactions were separated into two steps: uS8 synthesis and 16S rRNA transcription were performed in the first step (2 h) in the absence of recombinant ribosomal proteins, and then sfGFP template DNA and recombinant ribosomal proteins were added in the second step (4 h). The increase in sfGFP fluorescence after the 4-h incubation in the second step was normalized by dividing by the average value of the control reaction. Error bars indicate standard deviation of at least triplicate measurements.
