# Supplementary material for: In vitro reconstitution of functional small ribosomal subunit assembly for comprehensive analysis of ribosomal elements in E. coli
Source: Commun Biol. 2020 Mar 25;3:142. doi: 10.1038/s42003-020-0874-8 (PMC7096426; doi:10.1038/s42003-020-0874-8)
Supplement: Supplementary file 2 — Supplementary Data Descriptions [file 42003_2020_874_MOESM2_ESM.pdf]

Supplementary Data

Supplementary Data 1. Mass spectrometric analysis of protein mixtures used in R-iSAT. Twenty-four mixtures were analyzed by liquid chromatography–mass spectrometry, including ribosomal protein mixtures containing all of the recombinant ribosomal proteins from uS2 to bS21 and those without any specified ribosomal protein, a solution of bS1, a solution of 50S subunits, and solution 2 from PUREfrex 2.0 (GeneFrontier Corporation). After proteome analysis using Proteome Discoverer 2.2 (Thermo Fisher Scientific) to select peptides specific for each ribosomal protein, peptides with sufficient peptide spectrum matches (two to five peptides for each protein) were selected (peptides shown in red in each sheet) and quantified using Skyline (v4.2.0.18305; MacCoss Lab Software). Peak areas were calculated by setting MS1 filter to a count of three (M, M+1, and M+2).

Supplementary Data 2. DNA sequences of the plasmids used in preparation of DNA templates for R-iSAT. All plasmids were prepared by modifying the sequences between T7 promoter and T7 terminator in pET15b, pET32b, or pET26b (Merck Millipore, USA). Only a gene for ribosomal protein bS1 was cloned into pQE30 (QIAGEN, Germany) between T5 promoter and lambda t0 terminator. The modified regions between the promoter and the terminator are shown with visual annotations: Promoter and terminator regions are underlined; genes for each rRNA or protein are highlighted with gray; mutated regions from native sequences are shown with red characters. Note that the start codon of ribosomal protein uS13 and bS20 are encoded as GTG and TTG, respectively, in *E. coli* genome.

Supplementary Data 3. DNA primers used in PCR amplification of DNA templates for R-iSAT. Each DNA templates were amplified by 1- or 2-step PCR from plasmids shown in **Supplementary Data 2**.

Supplementary Data 4. Source data for main figures.
