# Supplementary material for: In vitro reconstitution of functional small ribosomal subunit assembly for comprehensive analysis of ribosomal elements in E. coli
Source: Commun Biol. 2020 Mar 25;3:142. doi: 10.1038/s42003-020-0874-8 (PMC7096426; doi:10.1038/s42003-020-0874-8)
Supplement: Supplementary file 5 — Supplementary Data 2 [file 42003_2020_874_MOESM5_ESM.pdf]

Supplementary Data 2. DNA sequences of the plasmids used in preparation of DNA templates for R-iSAT.

DNA sequences of the plasmids for *in vitro* 16S rRNA expression

>16S rRNA-wt in pET15b
TAATACGACTCACTATAGGGAAATTGAAGAGTTTGATCATGGCTCAGATTGAACGCTGGCGGCAGGCCTAACA
CATGCAAGTCGAACGGTAACAGGAAGAAGCTTGCTTCTTTGCTGACGAGTGGCGGACGGGTGAGTAATGTCTG
GGAAACTGCCTGATGGAGGGGGATAACTACTGGAAACGGTAGCTAATACCGCATAACGTCGCAAGACCAAAGA
GGGGGACCTTCGGGCCTCTTGCCATCGGATGTGCCCAGATGGGATTAGCTAGTAGGTGGGGTAACGGCTCACC
TAGGCGACGATCCCTAGCTGGTCTGAGAGGATGACCAGCCACACTGGAACTGAGACACGGTCCAGACTCCTAC
GGGAGGCAGCAGTGGGGAATATTGCACAATGGGCGCAAGCCTGATGCAGCCATGCCGCGTGTATGAAGAAGGC
CTTCGGGTTGTAAAGTACTTTCAGCGGGGAGGAAGGGAGTAAAGTTAATACCTTTGCTCATTGACGTTACCCG
CAGAAGAAGCACCGGCTAACTCCGTGCCAGCAGCCGCGGTAATACGGAGGGTGCAAGCGTTAATCGGAATTAC
TGGGCGTAAAGCGCACGCAGGCGGTTTGTTAAGTCAGATGTGAAATCCCCGGGCTCAACCTGGGAACTGCATC
TGATACTGGCAAGCTTGAGTCTCGTAGAGGGGGGTAGAATTCCAGGTGTAGCGGTGAAATGCGTAGAGATCTG
GAGGAATACCGGTGGCGAAGGCGGCCCCCTGGACGAAGACTGACGCTCAGGTGCGAAAGCGTGGGGAGCAAAC
AGGATTAGATACCCTGGTAGTCCACGCCGTAAACGATGTCGACTTGGAGGTTGTGCCCTTGAGGCGTGGCTTC
CGGAGCTAACGCGTTAAGTCGACCGCCTGGGGAGTACGGCCGCAAGGTTAAAACTCAAATGAATTGACGGGGG
CCCGCACAAGCGGTGGAGCATGTGGTTTAATTCGATGCAACGCGAAGAACCTTACCTGGTCTTGACATCCACG
GAAGTTTTCAGAGATGAGAATGTGCCTTCGGGAACCGTGAGACAGGTGCTGCATGGCTGTCGTCAGCTCGTGT
TGTGAAATGTTGGGTTAAGTCCCGCAACGAGCGCAACCCTTATCCTTTGTTGCCAGCGGTCCGGCCGGGAACT
CAAAGGAGACTGCCAGTGATAAACTGGAGGAAGGTGGGGATGACGTCAAGTCATCATGGCCCTTACGACCAGG
GCTACACACGTGCTACAATGGCGCATACAAAGAGAAGCGACCTCGCGAGAGCAAGCGGACCTCATAAAGTGCG
TCGTAGTCCGGATTGGAGTCTGCAACTCGACTCCATGAAGTCGGAATCGCTAGTAATCGTGGATCAGAATGCC
ACGGTGAATACGTTCCCGGGCCTTGTACACACCGCCCGTCACACCATGGGAGTGGGTTGCAAAAGAAGTAGGT
AGCTTAACCTTCGGGAGGGCGCTTACCACTTTGTGATTCATGACTGGGGTGAAGTCGTAACAAGGTAACCGTA
GGGGAACCTGCGGTTGGATCACCTCCTTACTAGCATAACCCCTTGGGGCCTCTAAACGGGTCTTGAGGGGTTT
TTTG

>16S rRNA-2 in pET15b
TAATACGACTCACTATAGGGAAATTGAAGAGTTTGATCATGGCTCAGATTGAACGCTGGCGGCAGGCCTAACA
CATGCAAGTCGAACGGTAACAGGAAGAAGCTTGCTTCTTTGCTGACGAGTGGCGGACGGGTGAGTAATGTCTG
GGAAACTGCCTGATGGAGGGGGATAACTACTGGAAACGGTAGCTAATACCGCATAACGTCGCAAGACCAAAGA
GGGGGACCTTCGGGCCTCTTGCCATCGGATGTGCCCAGATGGGATTAGCTAGTAGGTGGGGTAACGGCTCACC
TAGGCGACGATCCCTAGCTGGTCTGAGAGGATGACCAGCCACACTGGAACTGAGACACGGTCCAGACTCCTAC
GGGAGGCAGCAGTGGGGAATATTGCACAATGGGCGCAAGCCTGATGCAGCCATGCCGCGTGTATGAAGAAGGC
CTTCGGGTTGTAAAGTACTTTCAGCGGGGAGGAAGGGAGTAAAGTTAATACCTTTGCTCATTGACGTTACCCG
CAGAAGAAGCACCGGCTAACTCCGTGCCAGCAGCCGCGGTAATACGGAGGGTGCAAGCGTTAATCGGAATTAC
TGGGCGTAAAGCGCACGCAGGCGGTTTGTTAAGTCAGATGTGAAATCCCCGGGCTCAACCTGGGAACTGCATC

TGATACTGGCAAGCTTGAGTCTCGTAGAGGGGGGTAGAATTCCAGGTGTAGCGGTGAAATGCGTAGAGATCTG
GAGGAATACCGCGGGCGAAGGCGGCCCCCTGGACGAAGACTGACGCTCAGGTGCGAAAGCGTGGGGAGCAAAC
AGGATTAGATACCCTGGTAGTCCACGCCGTAAACGATGTCGACTTGGAGGTTGTGCCCTTGAGGCGTGGCTTC
CGGAGCTAACGCGTTAAGTCGACCGCCTGGGGAGTACGGCCGCAAGGTTAAAACTCAAATGAATTGACGGGGG
CCCGCACAAGCGGTGGAGCATGTGGTTTAATTCGATGCAACGCGAAGAACCTTACCTGGTCTTGACATCCACG
GAAGTTTTCAGAGATGAGAATGTGCCTTCGGGAACCGTGAGACAGGTGCTGCATGGCTGTCGTCAGCTCGTGT
TGTGAAATGTTGGGTTAAGTCCCGCAACGAGCGCAACCCTTATCCTTTGTTGCCAGCGGTCCGGCCGGGAACT
CAAAGGAGACTGCCAGTGATAAACTGGAGGAAGGTGGGGATGATGTCAAGTCATCATGGCCCTTACGACCAGG
GCTACACACGTGCTACAATGGCGCATACAAAGAGAAGCGACCTCGCGAGAGCAAGCGGACCTCATAAAGTGCG
TCGTAGTCCGGATTGGAGTCTGCAACTCGACTCCATGAAGTCGGAATCGCTAGTAATCGTGGATCAGAATGCC
ACGGTGAATACGTTCCCGGGCCTTGTACACACCGCCCGTCACACCATGGGAGTGGGTTGCAAAAGAAGTAGGT
AGCTTAACCTTCGGGAGGGCGCTTACCACTTTGTGATTCATGACTGGGGTGAAGTCGTAACAAGGTAACCGTA
GGGGAACCTGCGGTTGGATCACTGTGGTACTAGCATAACCCCTTGGGGCCTCTAAACGGGTCTTGAGGGGTTT
TTTG

>16S rRNA-8 in pET15b
TAATACGACTCACTATAGGGAAATTGAAGAGTTTGATCATGGCTCAGATTGAACGCTGGCGGCAGGCCTAACA
CATGCAAGTCGAACGGTAACAGGAAGAAGCTTGCTTCTTTGCTGACGAGTGGCGGACGGGTGAGTAATGTCTG
GGAAACTGCCTGATGGAGGGGGATAACTACTGGAAACGGTAGCTAATACCGCATAACGTCGCAAGACCAAAGA
GGGGGACCTTCGGGCCTCTTGCCATCGGATGTGCCCAGATGGGATTAGCTAGTAGGTGGGGTAACGGCTCACC
TAGGCGACGATCCCTAGCTGGTCTGAGAGGATGACCAGCCACACTGGAACTGAGACACGGTCCAGACTCCTAC
GGGAGGCAGCAGTGGGGAATATTGCACAATGGGCGCAAGCCTGATGCAGCCATGCCGCGTGTATGAAGAAGGC
CTTCGGGTTGTAAAGTACTTTCAGCGGGGAGGAAGGGAGTAAAGTTAATACCTTTGCTCATTGACGTTACCCG
CAGAAGAAGCACCGGCTAACTCCGTGCCAGCAGCCGCGGTAATACGGAGGGTGCAAGCGTTAATCGGAATTAC
TGGGCGTAAAGCGCACGCAGGCGGTTTGTTAAGTCAGATGTGAAATCCCCGGGCTCAACCTGGGAACTGCATC
TGATACTGGCAAGCTTGAGTCTCGTAGAGGGGGGTAGAATTCCAGGTGTAGCGGTGAAATGCGTAGAGATCTG
GAGGAATACCGCAGGCGAAGGCGGCCCCCTGGACGAAGACTGACGCTCAGGTGCGAAAGCGTGGGGAGCAAAC
AGGATTAGATACCCTGGTAGTCCACGCCGTAAACGATGTCGACTTGGAGGTTGTGCCCTTGAGGCGTGGCTTC
CGGAGCTAACGCGTTAAGTCGACCGCCTGGGGAGTACGGCCGCAAGGTTAAAACTCAAATGAATTGACGGGGG
CCCGCACAAGCGGTGGAGCATGTGGTTTAATTCGATGCAACGCGAAGAACCTTACCTGGTCTTGACATCCACG
GAAGTTTTCAGAGATGAGAATGTGCCTTCGGGAACCGTGAGACAGGTGCTGCATGGCTGTCGTCAGCTCGTGT
TGTGAAATGTTGGGTTAAGTCCCGCAACGAGCGCAACCCTTATCCTTTGTTGCCAGCGGTCCGGCCGGGAACT
CAAAGGAGACTGCCAGTGATAAACTGGAGGAAGGTGGGGATGATGTCAAGTCATCATGGCCCTTACGACCAGG
GCTACACACGTGCTACAATGGCGCATACAAAGAGAAGCGACCTCGCGAGAGCAAGCGGACCTCATAAAGTGCG
TCGTAGTCCGGATTGGAGTCTGCAACTCGACTCCATGAAGTCGGAATCGCTAGTAATCGTGGATCAGAATGCC
ACGGTGAATACGTTCCCGGGCCTTGTACACACCGCCCGTCACACCATGGGAGTGGGTTGCAAAAGAAGTAGGT
AGCTTAACCTTCGGGAGGGCGCTTACCACTTTGTGATTCATGACTGGGGTGAAGTCGTAACAAGGTAACCGTA
GGGGAACCTGCGGTTGGATCACCGCAGTACTAGCATAACCCCTTGGGGCCTCTAAACGGGTCTTGAGGGGTTT
TTTG

>16S rRNA-9 in pET15b
TAATACGACTCACTATAGGGAAATTGAAGAGTTTGATCATGGCTCAGATTGAACGCTGGCGGCAGGCCTAACA
CATGCAAGTCGAACGGTAACAGGAAGAAGCTTGCTTCTTTGCTGACGAGTGGCGGACGGGTGAGTAATGTCTG

GGAAACTGCCTGATGGAGGGGGATAACTACTGGAAACGGTAGCTAATACCGCATAACGTCGCAAGACCAAAGA
GGGGGACCTTCGGGCCTCTTGCCATCGGATGTGCCCAGATGGGATTAGCTAGTAGGTGGGGTAACGGCTCACC
TAGGCGACGATCCCTAGCTGGTCTGAGAGGATGACCAGCCACACTGGAACTGAGACACGGTCCAGACTCCTAC
GGGAGGCAGCAGTGGGGAATATTGCACAATGGGCGCAAGCCTGATGCAGCCATGCCGCGTGTATGAAGAAGGC
CTTCGGGTTGTAAAGTACTTTCAGCGGGGAGGAAGGGAGTAAAGTTAATACCTTTGCTCATTGACGTTACCCG
CAGAAGAAGCACCGGCTAACTCCGTGCCAGCAGCCGCGGTAATACGGAGGGTGCAAGCGTTAATCGGAATTAC
TGGGCGTAAAGCGCACGCAGGCGGTTTGTTAAGTCAGATGTGAAATCCCCGGGCTCAACCTGGGAACTGCATC
TGATACTGGCAAGCTTGAGTCTCGTAGAGGGGGGTAGAATTCCAGGTGTAGCGGTGAAATGCGTAGAGATCTG
GAGGAATACCGCAGGCGAAGGCGGCCCCCTGGACGAAGACTGACGCTCAGGTGCGAAAGCGTGGGGAGCAAAC
AGGATTAGATACCCTGGTAGTCCACGCCGTAAACGATGTCGACTTGGAGGTTGTGCCCTTGAGGCGTGGCTTC
CGGAGCTAACGCGTTAAGTCGACCGCCTGGGGAGTACGGCCGCAAGGTTAAAACTCAAATGAATTGACGGGGG
CCCGCACAAGCGGTGGAGCATGTGGTTTAATTCGATGCAACGCGAAGAACCTTACCTGGTCTTGACATCCACG
GAAGTTTTCAGAGATGAGAATGTGCCTTCGGGAACCGTGAGACAGGTGCTGCATGGCTGTCGTCAGCTCGTGT
TGTGAAATGTTGGGTTAAGTCCCGCAACGAGCGCAACCCTTATCCTTTGTTGCCAGCGGTCCGGCCGGGAACT
CAAAGGAGACTGCCAGTGATAAACTGGAGGAAGGTGGGGATGATGTCAAGTCATCATGGCCCTTACGACCAGG
GCTACACACGTGCTACAATGGCGCATACAAAGAGAAGCGACCTCGCGAGAGCAAGCGGACCTCATAAAGTGCG
TCGTAGTCCGGATTGGAGTCTGCAACTCGACTCCATGAAGTCGGAATCGCTAGTAATCGTGGATCAGAATGCC
ACGGTGAATACGTTCCCGGGCCTTGTACACACCGCCCGTCACACCATGGGAGTGGGTTGCAAAAGAAGTAGGT
AGCTTAACCTTCGGGAGGGCGCTTACCACTTTGTGATTCATGACTGGGGTGAAGTCGTAACAAGGTAACCGTA
GGGGAACCTGCGGTTGGATCATGGGATTACTAGCATAACCCCTTGGGGCCTCTAAACGGGTCTTGAGGGGTTT
TTTG

DNA sequence of the plasmid for *in vitro* sfGFP expression

>sfGFP in pET32b
TAATACGACTCACTATAGGGAGACCACAACGGTTTCCCTCTAGAAATAATTTTGTTTAACTTTAAGAAGGAGA
TATACCAATGAGTAAAGGAGAAGAACTTTTCACTGGAGTTGTCCCAATTCTTGTTGAATTAGATGGTGATGTT
AATGGGCACAAATTTTCTGTCCGTGGAGAGGGTGAAGGTGATGCAACAAACGGAAAACTTACCCTTAAATTTA
TTTGCACTACTGGAAAACTACCTGTTCCATGGCCAACACTTGTCACTACTTTAACTTATGGTGTTCAATGCTT
TTCCCGTTATCCGGATCACATGAAACGGCATGACTTTTTCAAGAGTGCCATGCCCGAAGGTTATGTACAGGAA
CGCACTATATCTTTCAAAGATGACGGGACCTACAAGACGCGTGCTGAAGTCAAGTTTGAAGGTGATACCCTTG
TTAATCGTATCGAGTTAAAAGGTATTGATTTTAAAGAAGATGGAAACATTCTCGGACACAAACTCGAGTACAA
CTTTAACTCACACAATGTATACATCACGGCAGACAAACAAAAGAATGGAATCAAAGCTAACTTCAAAATTCGC
CACAACGTTGAAGATGGATCCGTTCAACTAGCAGACCATTATCAACAAAATACTCCAATTGGCGATGGCCCTG
TCCTTTTACCAGACAACCATTACCTGTCGACACAATCTGTCCTTTCGAAAGATCCCAACGAAAAGCGTGACCA
CATGGTCCTTCTTGAGTTTGTAACTGCTGCTGGGATTACACATGGCATGGATGAGCTCTACAAATAATGAATT
CGAGCTCCGTCGACAAGCTTGCGGCCGCACTCGAGCATCACCATCACCATCACTAATGAATAACTAATCCCTA
GCATAACCCCTTGGGGCCTCTAAACGGGTCTTGAGGGGTTTTTTG

DNA sequences of the plasmids for *in vitro* ribosomal protein expression

>ribosomal protein bS1 in pQE30
TCATAAAAAATTTATTTGCTTTGTGAGCGGATAACAATTATAATAGATTCAATTGTGAGCGGATAACAATTTC
ACACAGAATTCATTAAAGAGGAGAAATTAACTATGAGAGGATCGCATCACCATCACCATCACGGATCCACTGA
ATCTTTTGCTCAACTCTTTGAAGAGTCCTTAAAAGAAATCGAAACCCGCCCGGGTTCTATCGTTCGTGGCGTT
GTTGTTGCTATCGACAAAGACGTAGTACTGGTTGACGCTGGTCTGAAATCTGAGTCCGCCATCCCGGCTGAGC
AGTTCAAAAACGCCCAGGGCGAGCTGGAAATCCAGGTAGGTGACGAAGTTGACGTTGCTCTGGACGCAGTAGA
AGACGGCTTCGGTGAAACTCTGCTGTCCCGTGAGAAAGCTAAACGTCACGAAGCCTGGATCACGCTGGAAAAA
GCTTACGAAGATGCTGAAACTGTTACCGGTGTTATCAACGGCAAAGTTAAGGGCGGCTTCACTGTTGAGCTGA
ACGGTATTCGTGCGTTCCTGCCAGGTTCTCTGGTAGACGTTCGTCCGGTGCGTGACACTCTGCACCTGGAAGG
CAAAGAGCTTGAATTTAAAGTAATCAAGCTGGATCAGAAGCGCAACAACGTTGTTGTTTCTCGTCGTGCCGTT
ATCGAATCCGAAAACAGCGCAGAGCGCGATCAGCTGCTGGAAAACCTGCAGGAAGGCATGGAAGTTAAAGGTA
TCGTTAAGAACCTCACTGACTACGGTGCATTCGTTGATCTGGGCGGCGTTGACGGCCTGCTGCACATCACTGA
CATGGCCTGGAAACGCGTTAAGCATCCGAGCGAAATCGTCAACGTGGGCGACGAAATCACTGTTAAAGTGCTG
AAGTTCGACCGCGAACGTACCCGTGTATCCCTGGGCCTGAAACAGCTGGGCGAAGATCCGTGGGTAGCTATCG
CTAAACGTTATCCGGAAGGTACCAAACTGACTGGTCGCGTGACCAACCTGACCGACTACGGCTGCTTCGTTGA
AATCGAAGAAGGCGTTGAAGGCCTGGTACACGTTTCCGAAATGGACTGGACCAACAAAAACATCCACCCGTCC
AAAGTTGTTAACGTTGGCGATGTAGTGGAAGTTATGGTTCTGGATATCGACGAAGAACGTCGTCGTATCTCCC
TGGGTCTGAAACAGTGCAAAGCTAACCCGTGGCAGCAGTTCGCGGAAACCCACAACAAGGGCGACCGTGTTGA
AGGTAAAATCAAGTCTATCACTGACTTCGGTATCTTCATCGGCTTGGACGGCGGCATCGACGGCCTGGTTCAC
CTGTCTGACATCTCCTGGAACGTTGCAGGCGAAGAAGCAGTTCGTGAATACAAAAAAGGCGACGAAATCGCTG
CAGTTGTTCTGCAGGTTGACGCAGAACGTGAACGTATCTCCCTGGGCGTTAAACAGCTCGCAGAAGATCCGTT
CAACAACTGGGTTGCTCTGAACAAGAAAGGCGCTATCGTAACCGGTAAAGTAACTGCAGTTGACGCTAAAGGC
GCAACCGTAGAACTGGCTGACGGCGTTGAAGGTTACCTGCGTGCTTCTGAAGCATCCCGTGACCGCGTTGAAG
ACGCTACCCTGGTTCTGAGCGTTGGCGACGAAGTTGAAGCTAAATTCACCGGCGTTGATCGTAAAAACCGCGC
AATCAGCCTGTCTGTTCGTGCGAAAGACGAAGCTGACGAGAAAGATGCAATCGCAACTGTTAACAAACAGGAA
GATGCAAACTTCTCCAACAACGCAATGGCTGAAGCTTTCAAAGCAGCTAAAGGCGAGTAAGTCGACCTGCAGC
CAAGCTTAATTAGCTGAGCTTGGACTCCTGTTGATAGATCCAGTAATGACCTCAGAACTCCATCTGGATTTGT
TCAGAACGCTCGGTTGCCGCCGGGCGTTTTTTATTGGTGAGAAT

>ribosomal protein uS2 in pET26b
TAATACGACTCACTATAGGGAGACCACAACGGTTTCCCTCTAGAAATAATTTTGTTTAACTTTAAGAAGGAGA
TATACCAATGGCAACTGTTTCCATGCGCGACATGCTCAAGGCTGGTGTTCACTTCGGTCACCAGACCCGTTAC
TGGAACCCGAAAATGAAGCCGTTCATCTTCGGTGCGCGTAACAAAGTTCACATCATCAACCTTGAGAAAACTG
TACCGATGTTCAACGAAGCTCTGGCTGAACTGAACAAGATTGCTTCTCGCAAAGGTAAAATCCTTTTCGTTGG
TACTAAACGCGCTGCAAGCGAAGCGGTGAAAGACGCTGCTCTGAGCTGCGACCAGTTCTTCGTGAACCATCGC
TGGCTGGGCGGTATGCTGACTAACTGGAAAACCGTTCGTCAGTCCATCAAACGTCTGAAAGACCTGGAAACTC
AGTCTCAGGACGGTACTTTCGACAAGCTGACCAAGAAAGAAGCGCTGATGCGCACTCGTGAGCTGGAGAAACT
GGAAAACAGCCTGGGCGGTATCAAAGACATGGGCGGTCTGCCGGACGCTCTGTTTGTAATCGATGCTGACCAC
GAACACATTGCTATCAAAGAAGCAAACAACCTGGGTATTCCGGTATTTGCTATCGTTGATACCAACTCTGATC
CGGACGGTGTTGACTTCGTTATCCCGGGTAACGACGACGCAATCCGTGCTGTGACCCTGTACCTGGGCGCTGT
TGCTGCAACCGTACGTGAAGGCCGTTCTCAGGATCTGGCTTCCCAGGCGGAAGAAAGCTTCGTAGAAGCTGAG

TAATGAATAACTAATCCCTAGCATAACCCCTTGGGGCCTCTAAACGGGTCTTGAGGGGTTTTTTG

>ribosomal protein uS3 in pET26b
TAATACGACTCACTATAGGGAGACCACAACGGTTTCCCTCTAGAAATAATTTTGTTTAACTTTAAGAAGGAGA
TATACCAATGGGTCAGAAAGTACATCCTAATGGTATTCGCCTGGGTATTGTAAAACCATGGAACTCTACCTGG
TTTGCGAACACCAAAGAATTCGCTGACAACCTGGACAGCGATTTTAAAGTACGTCAGTACCTGACTAAGGAAC
TGGCTAAAGCGTCCGTATCTCGTATCGTTATCGAGCGTCCGGCTAAGAGCATCCGTGTAACCATTCACACTGC
TCGCCCGGGTATCGTTATCGGTAAAAAAGGTGAAGACGTAGAAAAACTGCGTAAGGTCGTAGCGGACATCGCT
GGCGTTCCTGCACAGATCAACATCGCCGAAGTTCGTAAGCCTGAACTGGACGCAAAACTGGTTGCTGACAGCA
TCACTTCTCAGCTGGAACGTCGCGTTATGTTCCGTCGTGCTATGAAGCGTGCTGTACAGAACGCAATGCGTCT
GGGCGCTAAAGGTATTAAAGTTGAAGTTAGCGGCCGTCTGGGCGGCGCGGAAATCGCACGTACCGAATGGTAC
CGCGAAGGTCGCGTACCGCTGCACACTCTGCGTGCTGACATCGACTACAACACCTCTGAAGCGCACACCACTT
ACGGTGTAATCGGCGTTAAAGTGTGGATCTTCAAAGGCGAGATCCTGGGTGGTATGGCTGCTGTTGAACAACC
GGAAAAACCGGCTGCTCAGCCTAAAAAGCAGCAGCGTAAAGGCCGTAAATAATGAATAACTAATCCCTAGCAT
AACCCCTTGGGGCCTCTAAACGGGTCTTGAGGGGTTTTTTG

>ribosomal protein uS4 in pET26b
TAATACGACTCACTATAGGGAGACCACAACGGTTTCCCTCTAGAAATAATTTTGTTTAACTTTAAGAAGGAGA
TATACCAATGGCAAGATATTTGGGTCCTAAGCTCAAGCTGAGCCGTCGTGAGGGCACCGACTTATTCCTTAAG
TCTGGCGTTCGCGCGATCGATACCAAGTGTAAAATTGAACAAGCTCCTGGCCAGCACGGTGCGCGTAAACCGC
GTCTGTCTGACTATGGTGTGCAGTTGCGTGAAAAGCAAAAAGTTCGCCGTATCTATGGTGTGCTGGAGCGTCA
GTTCCGTAACTACTACAAAGAAGCAGCACGTCTGAAAGGCAACACCGGTGAAAACCTGTTGGCTCTGCTGGAA
GGTCGTCTGGACAACGTTGTATACCGTATGGGCTTCGGTGCCACTCGTGCAGAAGCACGTCAGCTGGTTAGCC
ATAAAGCAATTATGGTAAACGGTCGTGTTGTTAACATCGCTTCTTATCAGGTTAGTCCGAATGACGTTGTAAG
CATTCGTGAGAAAGCGAAGAAGCAGTCTCGCGTGAAAGCCGCTCTGGAGCTGGCTGAGCAGCGTGAAAAGCCA
ACCTGGCTGGAAGTTGATGCTGGCAAGATGGAAGGTACGTTTAAGCGTAAGCCGGAGCGTTCTGATCTGTCTG
CGGACATTAACGAACACCTGATCGTCGAGCTTTACTCCAAGTAATGAATAACTAATCCCTAGCATAACCCCTT
GGGGCCTCTAAACGGGTCTTGAGGGGTTTTTTG

>ribosomal protein uS5 in pET26b
TAATACGACTCACTATAGGGAGACCACAACGGTTTCCCTCTAGAAATAATTTTGTTTAACTTTAAGAAGGAGA
TATACCAATGGCTCACATCGAAAAACAAGCTGGCGAACTGCAGGAAAAGCTGATCGCGGTAAACCGCGTATCT
AAAACCGTTAAAGGTGGTCGTATTTTCTCCTTCACAGCTCTGACTGTAGTTGGCGATGGTAACGGTCGCGTTG
GTTTTGGTTACGGTAAAGCGCGTGAAGTTCCAGCAGCGATCCAGAAAGCGATGGAAAAAGCCCGTCGCAATAT
GATTAACGTCGCGCTGAATAACGGCACTCTGCAACACCCTGTTAAAGGTGTTCACACGGGTTCTCGCGTATTC
ATGCAGCCGGCTTCCGAAGGTACCGGTATCATCGCCGGTGGTGCAATGCGCGCCGTTCTGGAAGTCGCTGGGG
TTCATAACGTTCTGGCTAAAGCCTATGGTTCCACCAACCCGATCAACGTGGTTCGTGCAACTATTGATGGCCT
GGAAAATATGAATTCTCCAGAAATGGTCGCTGCCAAGCGTGGTAAATCCGTTGAAGAAATTCTGGGGAAATAA
TGAATAACTAATCCCTAGCATAACCCCTTGGGGCCTCTAAACGGGTCTTGAGGGGTTTTTTG

>ribosomal protein bS6 in pET26b
TAATACGACTCACTATAGGGAGACCACAACGGTTTCCCTCTAGAAATAATTTTGTTTAACTTTAAGAAGGAGA
TATACCAATGCGTCATTACGAAATCGTTTTTATGGTCCATCCTGACCAGAGCGAACAGGTTCCGGGCATGATC

GAGCGCTACACTGCTGCCATCACTGGTGCAGAAGGCAAGATCCACCGTCTGGAAGACTGGGGCCGCCGTCAGC
TGGCTTACCCGATCAACAAACTGCACAAAGCACACTACGTTCTGATGAATGTTGAAGCTCCGCAGGAAGTGAT
CGATGAGCTGGAAACTACCTTCCGCTTCAACGATGCCGTTATCCGCAGCATGGTTATGCGTACCAAGCACGCT
GTTACCGAAGCATCTCCGATGGTTAAAGCGAAAGACGAGCGCCGTGAGCGTCGCGATGATTTCGCAAACGAAA
CCGCTGATGATGCTGAAGCTGGGGATTCTGAAGAGTAATGAATAACTAATCCCTAGCATAACCCCTTGGGGCC
TCTAAACGGGTCTTGAGGGGTTTTTTG

>ribosomal protein uS7 in pET26b
TAATACGACTCACTATAGGGAGACCACAACGGTTTCCCTCTAGAAATAATTTTGTTTAACTTTAAGAAGGAGA
TATACCAATGCCACGTCGTCGCGTCATTGGTCAGCGTAAAATTCTGCCGGATCCGAAGTTCGGATCAGAACTG
CTGGCTAAATTTGTAAATATCCTGATGGTAGATGGTAAAAAATCTACTGCTGAATCTATCGTATACAGCGCGC
TGGAGACCCTGGCTCAGCGCTCTGGTAAATCTGAACTGGAAGCATTCGAAGTAGCTCTCGAAAACGTGCGCCC
GACTGTAGAAGTTAAGTCTCGCCGCGTTGGTGGTTCTACTTATCAGGTACCAGTTGAAGTCCGTCCGGTTCGT
CGTAATGCTCTGGCAATGCGTTGGATCGTTGAAGCTGCTCGTAAACGCGGTGATAAATCCATGGCTCTGCGCC
TGGCGAACGAACTTTCTGATGCTGCAGAAAACAAAGGTACTGCAGTTAAGAAACGTGAAGACGTTCACCGTAT
GGCCGAAGCCAACAAGGCGTTCGCACACTACCGTTGGTTATCCCTTCGGAGTTTTAGTCACCAGGCGGGCGCT
TCCAGTAAGCAGCCCGCTTTGGGCTACTTAAATTGATGAATAACTAATCCCTAGCATAACCCCTTGGGGCCTC
TAAACGGGTCTTGAGGGGTTTTTTG

>ribosomal protein uS8 in pET26b
TAATACGACTCACTATAGGGAGACCACAACGGTTTCCCTCTAGAAATAATTTTGTTTAACTTTAAGAAGGAGA
TATACCAATGAGCATGCAAGATCCGATCGCGGATATGCTGACCCGTATCCGTAACGGTCAGGCCGCGAACAAA
GCTGCGGTCACCATGCCTTCCTCCAAGCTGAAAGTGGCAATCGCCAACGTGCTGAAGGAAGAAGGTTTTATTG
AAGATTTTAAAGTTGAAGGCGACACCAAGCCTGAACTGGAACTTACTCTGAAGTATTTCCAGGGCAAAGCTGT
TGTAGAAAGCATTCAGCGTGTCAGCCGCCCAGGTCTGCGCATCTATAAACGTAAAGATGAGCTGCCGAAAGTT
ATGGCGGGTCTGGGTATCGCAGTTGTTTCTACCTCTAAAGGTGTTATGACTGATCGTGCAGCGCGCCAGGCTG
GTCTTGGTGGCGAAATTATCTGCTACGTAGCCTAATGAATAACTAATCCCTAGCATAACCCCTTGGGGCCTCT
AAACGGGTCTTGAGGGGTTTTTTG

>ribosomal protein uS9 in pET26b
TAATACGACTCACTATAGGGAGACCACAACGGTTTCCCTCTAGAAATAATTTTGTTTAACTTTAAGAAGGAGA
TATACCAATGGCTGAAAATCAATACTACGGCACTGGTCGCCGCAAAAGTTCCGCAGCTCGCGTTTTCATCAAA
CCGGGCAACGGTAAAATCGTAATCAACCAACGTTCTCTGGAACAGTACTTCGGTCGTGAAACTGCCCGCATGG
TAGTTCGTCAGCCGCTGGAACTGGTCGACATGGTTGAGAAACTGGACCTGTACATCACCGTTAAAGGTGGTGG
TATCTCTGGTCAGGCTGGTGCGATCCGTCACGGTATCACCCGCGCTCTGATGGAATACGACGAGTCCCTGCGT
TCTGAACTGCGTAAAGCTGGCTTCGTTACTCGTGACGCTCGTCAGGTTGAACGTAAGAAAGTCGGTCTGCGTA
AAGCACGTCGTCGTCCGCAGTTCTCCAAACGTTAATGAATAACTAATCCCTAGCATAACCCCTTGGGGCCTCT
AAACGGGTCTTGAGGGGTTTTTTG

>ribosomal protein uS10 in pET26b
TAATACGACTCACTATAGGGAGACCACAACGGTTTCCCTCTAGAAATAATTTTGTTTAACTTTAAGAAGGAGA
TATACCAATGCAGAACCAAAGAATCCGTATCCGCCTGAAAGCGTTTGATCATCGTCTGATCGATCAAGCAACC
GCGGAAATCGTCGAGACTGCCAAGCGCACTGGTGCGCAGGTCCGTGGTCCGATCCCGCTGCCGACACGCAAAG

AGCGCTTCACTGTTCTGATCTCCCCGCACGTCAACAAAGACGCGCGCGATCAGTACGAAATCCGTACTCACTT
GCGTCTGGTTGACATCGTTGAGCCAACCGAGAAAACCGTTGATGCTCTGATGCGTCTGGATCTGGCTGCCGGT
GTAGACGTGCAGATCAGCCTGGGTTAATGAATAACTAATCCCTAGCATAACCCCTTGGGGCCTCTAAACGGGT
CTTGAGGGGTTTTTTG

>ribosomal protein uS11 in pET26b
TAATACGACTCACTATAGGGAGACCACAACGGTTTCCCTCTAGAAATAATTTTGTTTAACTTTAAGAAGGAGA
TATACCAATGGCAAAGGCACCAATTCGTGCACGTAAACGTGTAAGAAAACAAGTCTCTGACGGCGTGGCTCAT
ATCCATGCTTCTTTCAACAACACCATCGTGACTATCACTGATCGTCAGGGTAACGCGTTGGGTTGGGCAACAG
CCGGTGGTTCCGGTTTCCGTGGTTCTCGCAAATCCACTCCGTTTGCAGCTCAGGTTGCAGCAGAGCGTTGCGC
TGACGCCGTGAAAGAATACGGCATCAAGAATCTGGAAGTTATGGTTAAAGGTCCGGGTCCAGGCCGCGAATCT
ACTATTCGTGCTCTGAACGCCGCAGGTTTCCGCATCACTAACATTACTGATGTGACTCCGATCCCTCATAACG
GTTGTCGTCCGCCGAAAAAACGTCGCGTATAATGAATAACTAATCCCTAGCATAACCCCTTGGGGCCTCTAAA
CGGGTCTTGAGGGGTTTTTTG

>ribosomal protein uS12 in pET26b
TAATACGACTCACTATAGGGAGACCACAACGGTTTCCCTCTAGAAATAATTTTGTTTAACTTTAAGAAGGAGA
TATACCAATGGCAACAGTTAACCAGCTGGTACGCAAACCACGTGCTCGCAAAGTTGCGAAAAGCAACGTGCCT
GCGCTGGAAGCATGCCCGCAAAAACGTGGCGTATGTACTCGTGTATATACTACCACTCCTAAAAAACCGAACT
CCGCGCTGCGTAAAGTATGCCGTGTTCGTCTGACTAACGGTTTCGAAGTGACTTCCTACATCGGTGGTGAAGG
TCACAACCTGCAGGAGCACTCCGTGATCCTGATCCGTGGCGGTCGTGTTAAAGACCTCCCGGGTGTTCGTTAC
CACACCGTACGTGGTGCGCTTGACTGCTCCGGCGTTAAAGACCGTAAGCAGGCTCGTTCCAAGTATGGCGTGA
AGCGTCCTAAGGCTTAATGAATAACTAATCCCTAGCATAACCCCTTGGGGCCTCTAAACGGGTCTTGAGGGGT
TTTTTG

>ribosomal protein uS13 in pET26b
TAATACGACTCACTATAGGGAGACCACAACGGTTTCCCTCTAGAAATAATTTTGTTTAACTTTAAGAAGGAGA
TATACCAATGGCCCGTATAGCAGGCATTAACATTCCTGATCATAAGCATGCCGTAATCGCATTAACTTCGATT
TATGGCGTCGGCAAGACCCGTTCTAAAGCCATCCTGGCTGCAGCGGGTATCGCTGAAGATGTTAAGATCAGTG
AGCTGTCTGAAGGACAAATCGACACGCTGCGTGACGAAGTTGCCAAATTTGTCGTTGAAGGTGATCTGCGCCG
TGAAATCAGCATGAGCATCAAGCGCCTGATGGATCTTGGTTGCTATCGCGGTTTGCGTCATCGTCGTGGTCTC
CCGGTTCGCGGTCAGCGTACCAAGACCAACGCACGTACCCGTAAGGGTCCGCGCAAACCGATCAAGAAATAAT
GAATAACTAATCCCTAGCATAACCCCTTGGGGCCTCTAAACGGGTCTTGAGGGGTTTTTTG

>ribosomal protein uS14 in pET26b
TAATACGACTCACTATAGGGAGACCACAACGGTTTCCCTCTAGAAATAATTTTGTTTAACTTTAAGAAGGAGA
TATACCAATGGCTAAGCAATCAATGAAAGCACGCGAAGTAAAACGCGTAGCTTTAGCTGATAAATACTTCGCG
AAACGCGCTGAACTGAAAGCGATCATCTCTGATGTGAACGCTTCCGACGAAGATCGTTGGAACGCTGTTCTCA
AGCTGCAGACTCTGCCGCGTGATTCCAGCCCGTCTCGTCAGCGTAACCGCTGCCGTCAAACAGGTCGTCCGCA
TGGTTTCCTGCGGAAGTTCGGGTTGAGCCGTATTAAGGTCCGTGAAGCCGCTATGCGCGGTGAAATCCCGGGT
CTGAAAAAGGCTAGCTGGTAATGAATAACTAATCCCTAGCATAACCCCTTGGGGCCTCTAAACGGGTCTTGAG
GGGTTTTTTG

>ribosomal protein uS15 in pET26b
TAATACGACTCACTATAGGGAGACCACAACGGTTTCCCTCTAGAAATAATTTTGTTTAACTTTAAGAAGGAGA
TATACCAATGTCTCTAAGTACTGAAGCAACAGCTAAAATCGTTTCTGAGTTTGGTCGTGACGCAAACGACACC
GGTTCTACCGAAGTTCAGGTAGCACTGCTGACTGCACAGATCAACCACCTGCAGGGCCACTTTGCAGAGCACA
AAAAAGATCACCACAGCCGTCGTGGTCTGCTGCGCATGGTTTCTCAGCGTCGTAAACTGCTCGACTACCTGAA
ACGTAAAGACGTAGCACGTTACACCCAGCTCATCGAGCGCCTGGGTCTGCGTCGCTAATGAATAACTAATCCC
TAGCATAACCCCTTGGGGCCTCTAAACGGGTCTTGAGGGGTTTTTTG

>ribosomal protein bS16 in pET26b
TAATACGACTCACTATAGGGAGACCACAACGGTTTCCCTCTAGAAATAATTTTGTTTAACTTTAAGAAGGAGA
TATACCAATGGTAACTATTCGTTTAGCACGTCACGGCGCTAAAAAGCGTCCGTTCTACCAGGTTGTTGTCGCT
GACAGCCGTAATGCACGCAACGGTCGCTTCATCGAGCGCGTTGGTTTCTTCAACCCAATCGCTAGCGAAAAAG
AAGAAGGCACTCGCCTGGATCTGGATCGCATCGCTCACTGGGTTGGCCAGGGCGCAACTATTTCTGATCGCGT
TGCTGCGCTGATCAAAGAAGTAAACAAAGCAGCTTAATGAATAACTAATCCCTAGCATAACCCCTTGGGGCCT
CTAAACGGGTCTTGAGGGGTTTTTTG

>ribosomal protein uS17 in pET26b
TAATACGACTCACTATAGGGAGACCACAACGGTTTCCCTCTAGAAATAATTTTGTTTAACTTTAAGAAGGAGA
TATACCAATGACCGATAAAATCCGTACTCTGCAAGGTCGCGTTGTTAGCGACAAAATGGAGAAATCCATTGTT
GTTGCTATCGAACGTTTTGTGAAACACCCGATCTACGGTAAATTCATCAAGCGTACGACCAAACTGCACGTAC
ATGACGAGAACAACGAATGCGGTATCGGTGACGTGGTTGAAATCCGCGAATGCCGTCCGCTGTCCAAGACTAA
ATCCTGGACGCTGGTTCGCGTTGTAGAGAAAGCGGTTCTGTAATGAATAACTAATCCCTAGCATAACCCCTTG
GGGCCTCTAAACGGGTCTTGAGGGGTTTTTTG

>ribosomal protein bS18 in pET26b
TAATACGACTCACTATAGGGAGACCACAACGGTTTCCCTCTAGAAATAATTTTGTTTAACTTTAAGAAGGAGA
TATACCAATGGCACGTTATTTCCGTCGTCGCAAGTTCTGCCGTTTCACCGCGGAAGGCGTTCAAGAGATCGAC
TATAAAGATATCGCTACGCTGAAAAACTACATCACCGAAAGCGGTAAGATTGTCCCAAGCCGTATCACCGGTA
CCCGTGCAAAATACCAGCGTCAGCTGGCTCGCGCTATCAAACGCGCTCGCTACCTGTCCCTGCTGCCGTACAC
TGATCGCCATCAGTAATGAATAACTAATCCCTAGCATAACCCCTTGGGGCCTCTAAACGGGTCTTGAGGGGTT
TTTTG

>ribosomal protein uS19 in pET26b
TAATACGACTCACTATAGGGAGACCACAACGGTTTCCCTCTAGAAATAATTTTGTTTAACTTTAAGAAGGAGA
TATACCAATGCCACGTTCTCTCAAGAAAGGTCCTTTTATTGACCTGCACTTGCTGAAGAAGGTAGAGAAAGCG
GTGGAAAGCGGAGACAAGAAGCCCCTGCGCACTTGGTCCCGTCGTTCAACGATCTTTCCTAACATGATCGGTT
TGACCATCGCTGTCCATAATGGTCGTCAGCACGTTCCGGTATTTGTAACCGACGAAATGGTTGGTCACAAACT
GGGTGAATTCGCACCGACTCGTACTTATCGCGGCCACGCTGCTGATAAAAAAGCGAAGAAGAAATAATGAATA
ACTAATCCCTAGCATAACCCCTTGGGGCCTCTAAACGGGTCTTGAGGGGTTTTTTG

>ribosomal protein bS20 in pET26b
TAATACGACTCACTATAGGGAGACCACAACGGTTTCCCTCTAGAAATAATTTTGTTTAACTTTAAGAAGGAGA
TATACCAATGGCTAATATCAAATCAGCTAAGAAGCGCGCCATTCAGTCTGAAAAGGCTCGTAAGCACAACGCA

AGCCGTCGCTCTATGATGCGTACTTTCATCAAGAAAGTATACGCAGCTATCGAAGCTGGCGACAAAGCTGCTG
CACAGAAAGCATTTAACGAAATGCAACCGATCGTGGACCGTCAGGCTGCTAAAGGTCTGATCCACAAAAACAA
AGCTGCACGTCATAAGGCTAACCTGACTGCACAGATCAACAAACTGGCTTAATGAATAACTAATCCCTAGCAT
AACCCCTTGGGGCCTCTAAACGGGTCTTGAGGGGTTTTTTG

>ribosomal protein bS21 in pET26b
TAATACGACTCACTATAGGGAGACCACAACGGTTTCCCTCTAGAAATAATTTTGTTTAACTTTAAGAAGGAGA
TATACCAATGCCGGTAATTAAAGTACGTGAAAACGAGCCGTTCGACGTAGCTCTGCGTCGCTTCAAGCGTTCC
TGCGAAAAAGCAGGTGTTCTGGCGGAAGTTCGTCGTCGTGAGTTCTATGAAAAACCGACTACCGAACGTAAGC
GCGCTAAAGCTTCTGCAGTGAAACGTCACGCGAAGAAACTGGCTCGCGAAAACGCACGCCGCACTCGTCTGTA
CTAATGAATAACTAATCCCTAGCATAACCCCTTGGGGCCTCTAAACGGGTCTTGAGGGGTTTTTTG

>K42T mutant of ribosomal protein uS12 in pET26b
TAATACGACTCACTATAGGGAGACCACAACGGTTTCCCTCTAGAAATAATTTTGTTTAACTTTAAGAAGGAGA
TATACCAATGGCAACAGTTAACCAGCTGGTACGCAAACCACGTGCTCGCAAAGTTGCGAAAAGCAACGTGCCT
GCGCTGGAAGCATGCCCGCAAAAACGTGGCGTATGTACTCGTGTATATACTACCACTCCTACAAAACCGAACT
CCGCGCTGCGTAAAGTATGCCGTGTTCGTCTGACTAACGGTTTCGAAGTGACTTCCTACATCGGTGGTGAAGG
TCACAACCTGCAGGAGCACTCCGTGATCCTGATCCGTGGCGGTCGTGTTAAAGACCTCCCGGGTGTTCGTTAC
CACACCGTACGTGGTGCGCTTGACTGCTCCGGCGTTAAAGACCGTAAGCAGGCTCGTTCCAAGTATGGCGTGA
AGCGTCCTAAGGCTTAATGAATAACTAATCCCTAGCATAACCCCTTGGGGCCTCTAAACGGGTCTTGAGGGGT
TTTTTG

>P90L mutant of ribosomal protein uS12 in pET26b
TAATACGACTCACTATAGGGAGACCACAACGGTTTCCCTCTAGAAATAATTTTGTTTAACTTTAAGAAGGAGA
TATACCAATGGCAACAGTTAACCAGCTGGTACGCAAACCACGTGCTCGCAAAGTTGCGAAAAGCAACGTGCCT
GCGCTGGAAGCATGCCCGCAAAAACGTGGCGTATGTACTCGTGTATATACTACCACTCCTAAAAAACCGAACT
CCGCGCTGCGTAAAGTATGCCGTGTTCGTCTGACTAACGGTTTCGAAGTGACTTCCTACATCGGTGGTGAAGG
TCACAACCTGCAGGAGCACTCCGTGATCCTGATCCGTGGCGGTCGTGTTAAAGACCTCCTGGGTGTTCGTTAC
CACACCGTACGTGGTGCGCTTGACTGCTCCGGCGTTAAAGACCGTAAGCAGGCTCGTTCCAAGTATGGCGTGA
AGCGTCCTAAGGCTTAATGAATAACTAATCCCTAGCATAACCCCTTGGGGCCTCTAAACGGGTCTTGAGGGGT
TTTTTG

>G91D mutant of ribosomal protein uS12 in pET26b
TAATACGACTCACTATAGGGAGACCACAACGGTTTCCCTCTAGAAATAATTTTGTTTAACTTTAAGAAGGAGA
TATACCAATGGCAACAGTTAACCAGCTGGTACGCAAACCACGTGCTCGCAAAGTTGCGAAAAGCAACGTGCCT
GCGCTGGAAGCATGCCCGCAAAAACGTGGCGTATGTACTCGTGTATATACTACCACTCCTAAAAAACCGAACT
CCGCGCTGCGTAAAGTATGCCGTGTTCGTCTGACTAACGGTTTCGAAGTGACTTCCTACATCGGTGGTGAAGG
TCACAACCTGCAGGAGCACTCCGTGATCCTGATCCGTGGCGGTCGTGTTAAAGACCTCCCGGATGTTCGTTAC
CACACCGTACGTGGTGCGCTTGACTGCTCCGGCGTTAAAGACCGTAAGCAGGCTCGTTCCAAGTATGGCGTGA
AGCGTCCTAAGGCTTAATGAATAACTAATCCCTAGCATAACCCCTTGGGGCCTCTAAACGGGTCTTGAGGGGT
TTTTTG
